# Books and Magazines

**Published:** 1904-09

**Authors:** 


### Books and Magazines

---

A DICTIONARY OF MEDICAL SCIENCE, containing a full explanation of the various subjects and terms of Anatomy, Physiology, Chemistry, Pharmacy, Hygiene, Dietetics, Pathology, Ophthalmology, Otology, Dermatology, Gynecology, Obstetrics, Pediatrics, Medical Jurisprudence, Dentistry, and Veterinary Science, etc. By Robley Dunglison, M. D., LL. D., Late Professor in the Jefferson Medical College of Philadelphia. Twenty-third edition, thoroughly revised by Thos. L. Stedman, A. M., M. D., Fellow of New York Academy of Medicine. Published by Lea Bros. & Co., Philadelphia and New York. Price, $8.00.

In the preface to this work the revisor of the edition says that he ought to be spared the usual task of perfecting this work, for a book which has held its own for twenty-two editions, and nearly three-quarters of a century, is too well known by the profession to be criticised favorably or adversely by a single man. The reviewing editor agrees most emphatically with this idea. The Dictionary is thoroughly complete and elaborate, yet practically so, and in every sense holds its own as a book of medical reference. To say that the work should be in every doctor's library is to put it mildly. We prefer to say that the doctor who fails to have it is unfortunate.

DISEASES OF THE NOSE AND THROAT. By D. Braden Kyle, M. D., Professor of Laryngology and Rhinology, Jefferson Medical College, Philadelphia; consulting laryngologist, rhinologist and otologist, St. Agnes' Hospital. Third edition, thoroughly revised and enlarged. Octavo volume of 669 pages, with 175 illustrations, and 6 chromo-lithographic plates. Philadelphia, New

York, London: W. B. Saunders & Company, 1904. Cloth, $4, net; sheep or half Morocco, $5, net.

In presenting to the profession the third edition of this work the general plan of the previous editions has not been materially altered. The entire book has been carefully revised and such additions have been made as were rendered necessary by recent medical progress. The most important alterations and additions have been made in the chapters on Keratosis, Epidemic Influenza, Gersuny's Paraffine Method for the correction of Nasal Deformities, and in the one on the X-rays in the treatment of Carcinoma. The etiology and treatment of hay fever have been partially rewritten and much enlarged, as has also the operative treatment of deformities of the nasal septum. In the chapter devoted to general considerations of Mucous Membranes and Hay Fever the author records the results of his experience in the chemistry of the saliva and nasal secretions in relation to diagnosis and treatment. The literature has been carefully reviewed, and a number of new illustrations added, thus bringing the work absolutely down to date.

---

MEDICAL DIAGNOSIS.—SPECIAL DIAGNOSIS OF INTERNAL MEDICINE.—A HANDBOOK FOR PHYSICIANS AND STUDENTS. By Dr. Wilhelm v. Luebe, Professor of Medicine and Physician-in-chief to the Julius Hospital at Würzburg. Authorized translations from the sixth German edition. Edited, with annotations, by Julius L. Salinger, M. D., late assistant professor of Clinical Medicine in the Jefferson Medical College. With five colored plates and seventy-four illustrations in the text. Cloth. 1058 pages. Price, $5. New York, London: D. Appleton & Company, 1904.

Luebe's diagnosis has long been recognized as a standard work, and its translation from the sixth German edition is welcome. In general the translation has been well done, and the editor has made occasional valuable notations. Once in a while a too literal adherence to the German idiom is seen, as when "pulsions diverticulum" is referred to, and when that puzzling word "respective" is translated "respectively," e. g., "Subacidly respectively anacidity" (p. 295). In some of the illustrations there is a strange mixture of Latin and English terms. Thus, Figure 27, p. 514, we find not only "canalis centralia," "commissura anterior grisea," etc., but also "posterior horn," "lateral cornu." Of Leube's work little need be said. The book is full of Luebe's personalty, the experience of a man of judgment, who has had years of varied clinical experience. It is distinctly a practical work, though the theoretical is not ignored, and one may be sure that anything described by Leube is viewed from the scientific standpoint. It is needless to say that such diseases as those of the stomach, ulcer, for instance, a subject in which Leube was one of the pioneers, are described in a wonderfully complete yet compact form, as can be done by one who is fully master of his subject. We could pick flaws here and there, could wish that under epidemic meningitis, lumbar puncture and the Kernig sign were brought more to the front, that tricuspid stenosis, pylorus spasm, etc., were enlarged on a little more, but, take it for all in all, the work is quite a complete mine of information, imparted in a modest manner, and often helping out the perplexed practitioner just where he needs help, i. e., in the very places where Leube was perplexed some fifteen or twenty odd years ago, and which he now tells us about in a way both pleasing and instructive.—*Exchange.*

---

A Reference Handbook of the Medical Sciences, Embracing the Entire Range of Practical and Scientific Medicine and Allied Science. By various writers. A new edition, completely revised and re-written. Edited by Albert H. Buck, M. D., New York City. Volume VIII. William Wood & Co., New York, 1904.

We have before us the 8th and last volume of this masterpiece. The work in its entirety is the most complete and up-to-date publication of the kind extant. This volume gives a full interpretation, in addition to the subject matter proper, of terms in general use in medicine and surgery; and contains an appendix giving much valuable information gained from current medical literature since the manuscript was handed in. It is profusely illustrated with fine chromo lithographs and nearly 500 half-tone and wood engravings. The set is a desirable, nay indispensable, acquisition to all progressive physicians.

Let it be remembered that this is a new edition, completely revised and re-written, with many additions since the last edition was issued some years ago. Each of the eight volumes is a large quarto of some seven hundred or eight hundred pages. The mechanical work is high class, and does credit to the great publishing house of Wm. Wood & Co. Volume 8 runs from "Umb" to "Zym," and includes the appendix mentioned, which is alphabetically arranged. The article on Vital Statistics is by Dr. Samuel W. Abbott, the well known Secretary of the Massachusetts State Board of Health, and is alone worth the price of the set. It covers nineteen pages double column, and is complete and exhaustive.

Volume 8 also contains the general index of the set, double and cross entries, for quick and convenient reference to any volume and any subject.

---

The Doctor's Recreation Series. Charles Wells Moulton, general editor. Published by the Saalsfield Publishing Company, Akron, O. Facts and fancies of interest to the doctor and his patient. Arranged by Porter Davis, M. D. Price per volume, cloth, $2.50; leather, $4. 352 pages.

We have received Volume II of the series. It is entitled "The Doctor's Red Lamp." We don't exactly see the appropriateness of the name, but then, like the rose, it would be as sweet by any other name. I have read it through. It is good. It is very interesting. "A Will and a Way"—one story in it,—is worth many times the price of the book. If the editor keeps the standard so high all the way through, it will be a very valuable and desirable series; a library of wit and humor, with a sprinkle of the pathetic and also of the horrible. I await Volume 3 with impatience. By the bye, why not cut the leaves apart, and trim the edges? It is an abominable fad to leave them uncut, and a bore to have to cut them. Besides, rough edges get dirty so easy. There is no sense in it.

